# ERP Decoding Analysis of Visual Working Memory Processes in Schizophrenia

**DOI:** 10.31083/AP42597

**Published:** 2026-02-25

**Authors:** Zhongsi Wang, Chunlei Liu, Yuyan Jing, Zhenzhen Yao, Min Chen

**Affiliations:** ^1^School of Psychology, Qufu Normal University, 273165 Qufu, Shandong, China; ^2^School of Mental Health, Jining Medical University, 272000 Jining, Shandong, China; ^3^Department of Psychiatry, Shandong Daizhuang Hospital, 272051 Jining, Shandong, China

**Keywords:** decoding, event-related potential, schizophrenia, cognitive deficit, visual working memory

## Abstract

**Background:**

Deficits in visual working memory (vWM) represent a core cognitive impairment in schizophrenia; however, the dynamic spatiotemporal characterization of their underlying neural mechanisms remains unclear. The present study employed multivariate pattern classification (MVPC) and searchlight analysis to investigate neural signaling differences between patients with schizophrenia (PSZ) and healthy control subjects (HCS) during a vWM task.

**Methods:**

A total of 46 participants (22 PSZ, 24 HCS) completed a change detection task comprising three conditions: two targets, zero distractors (2T0D); two targets, two distractors (2T2D); and four targets, zero distractors (4T0D). Contralateral delay activity (CDA) was extracted through event-related potential (ERP) analysis. MVPC was applied in the temporal dimension, while a searchlight approach was employed in the spatial dimension to decode memory load (2T0D/2T2D/4T0D) and memory side (left/right) information.

**Results:**

CDA amplitude was significantly reduced in the PSZ group, particularly in the 2T2D condition (*p* = 0.01), indicating that the scope and control of attention elicited comparable CDA amplitudes. MVPC analysis revealed that decoding accuracy in the PSZ group was significantly lower than in the HCS group during the time window of 93–652 ms (*p*_corrected_ < 0.05), suggesting diminished efficiency of neural information encoding during the delay period. The searchlight analysis identified the most pronounced decrease in decoding accuracy within the left parietal region in the PSZ group, consistent with the hypothesis of abnormal functional connectivity in the inferior parietal gyrus (IPG).

**Conclusions:**

This study reveals the spatiotemporal dynamics of vWM deficits in schizophrenia, characterized by ERP decoding technology. It offers a novel target for the development of neuromarker-based cognitive interventions.

## Main Points

1. Patients with schizophrenia exhibit significantly reduced neural decoding accuracy during the visual working memory delay period compared to healthy controls, indicating diminished maintenance of stimulus-specific information in neural signals (93–652 ms post-stimulus).

2. Spatial decoding analysis reveals pronounced deficits in the left parietal cortex of patients with schizophrenia, with searchlight analysis showing significantly lower decoding accuracy across all electrode sites, particularly in left parietal regions, suggesting compromised neural information representation in this area.

3. Patients with schizophrenia demonstrate reduced contralateral delay activity amplitudes and lower working memory capacity, with the most severe behavioral and neural impairments observed under conditions requiring attention control (distractor presence), though they maintain above-chance decoding ability.

4. Multivariate pattern classification and searchlight decoding methods effectively reveal the spatiotemporal dynamics of visual working memory deficits in schizophrenia, providing a sensitive, individualized approach to characterizing neural representation impairments that extends beyond traditional univariate event-related potential (ERP) analyses.

## 1. Introduction

Schizophrenia is a complex and severe brain disorder characterized by neurocognitive dysfunction at its core [1]. According to the World Health Organization, by 2021, nearly 24 million people worldwide were projected to suffer from this disorder, resulting in a significant social burden. Impairment in working memory (WM) is a fundamental cognitive deficit associated with schizophrenia [2, 3, 4, 5] and has been identified as a potential warning sign for the onset of psychosis [6, 7, 8].

Since visual working memory (vWM) is strongly correlated with higher cognitive functioning, recent research on working memory in patients with schizophrenia (PSZ) has focused on vWM [9, 10, 11, 12, 13, 14, 15]. Attention plays a crucial role in the encoding, maintenance, and extraction of vWM. Based on their independent contributions to vWM performance, attention processes can be subdivided into scope and control [16, 17, 18, 19]. Attention scope refers to the quantity of information that can be actively maintained over a specific duration, while attention control refers to the ability to focus on relevant information while ignoring irrelevant details. Both attention scope and attention control significantly impact vWM capacity [16].

Contralateral delay activity (CDA), a negative slow wave sensitive to the number of objects held in vWM, is commonly used to examine neural activity during the maintenance phase of vWM. Its possible source has been localized to the posterior parietal cortex [20, 21, 22]. A key feature of CDA is that its amplitude increases with the number of objects held in vWM [23, 24, 25]. Numerous studies have demonstrated that PSZ exhibit smaller CDA amplitudes and reduced vWM capacity [10, 11, 14, 26].

Decoding methods are frequently employed to study neurocognitive processing mechanisms in typical individuals and to quantify the information contained in the neural signals of individual participants. Researchers gain insights into brain processing by making statistical inferences about the availability of information [27]. Additionally, comparing decoding accuracy across different brain regions and time points can help elucidate the location and time course of information processing in the brain [28]. In previous studies, researchers have predominantly applied decoding methods to functional magnetic resonance imaging (fMRI) studies, often overlooking the potential benefits of applying these methods to electroencephalogram (EEG) studies.

Multivariate pattern classification (MVPC) is a decoding method employed for EEG data. MVPC utilizes the scalp distribution of EEG signals to decode or track the information contained within these signals, enabling the assessment of how the informational content of neural signals evolves over time following the onset of stimulation [28]. This method has been successfully applied to identify the neural mapping between activity distribution patterns and corresponding mental states [29, 30, 31, 32]. For many inquiries regarding mental disorders—particularly in the domains of perception, attention, and working memory—temporal discrimination may be as crucial as spatial discrimination [33]. In cognitive neuroscience, searchlight analysis is extensively utilized to localize brain regions associated with specific mental processes [34]. Searchlight analysis is entirely data-driven and does not employ any geometric transformations to address high-dimensional problems, thereby preserving spatial information in its original context [35].

In vWM research, the change detection task has become a widely used method for estimating working memory capacity [24]. Therefore, the change detection task was selected as the experimental task for this study, with the CDA serving as the event-related potential (ERP) indicator. Two decoding methods—MVPC and searchlight analysis—were employed to decode the memory load and memory side of ERPs across temporal and spatial dimensions, respectively. The aim was to investigate the neural signaling differences between PSZ and healthy controls (HCS) during a vWM task.

## 2. Methods

### 2.1 Participants

According to G*Power 3.1.9.4 (https://www.psychologie.hhu.de/) calculations [36], repeated measures analyses of within-group and between-group interactions were conducted in the present study, with a statistical power of 95%, α= 0.05, and a medium effect size of f = 0.25, necessitating a minimum of 44 participants. 25 PSZ and 25 HCS were recruited for the current experiment. The PSZ were recruited from Daizhuang Hospital in Shandong Province by their attending physician. The patients were clinically stable and either not on medication or treated with low doses of atypical antipsychotics (less than 300 mg chlorpromazine equivalents). The HCS were healthy volunteers from the community surrounding Daizhuang Hospital in Shandong Province during the same time period. The HCS had no personal or familial history of psychiatric disorders in their first-degree relatives, nor any hereditary neurological disorders, and had not engaged in any behavioral habits affecting the central nervous system in the month prior to the study. One HCS was excluded due to an interruption in data recording, and 3 PSZ were excluded due to a high number of artifacts, with the remaining participants having fewer than 25% artifacts. Ultimately, a total of 46 participants were included in the analysis: 22 PSZ and 24 HCS.

Participant exclusion criteria included: (i) a history of traumatic brain injury, neurological disease, or other significant physical illnesses; (ii) prior receipt of electroconvulsive therapy; (iii) a history of alcohol or drug abuse or dependence; (iv) individuals with secondary psychotic symptoms resulting from other organic causes or substance use; and (v) intellectual disability.

### 2.2 Clinical and Functioning Assessments

The Positive and Negative Syndrome Scale (PANSS) [37], which is commonly used in clinical practice to assess the clinical symptoms of PSZ, was used. The PANSS consists of the General Psychopathology Scale (GPS), the Positive Symptoms Subscale (PSS), the Negative Symptoms Subscale (NSS), and three supplemental scales, totalling 33 items. The Spatial Span (SS) and Number Span (NS) of the MATRICS Consensus Cognitive Battery (MCCB) were selected for working memory assessment, and the change detection task was selected for assessing vWM [24].

### 2.3 Stimulus and Procedure

**Fig. 1.**
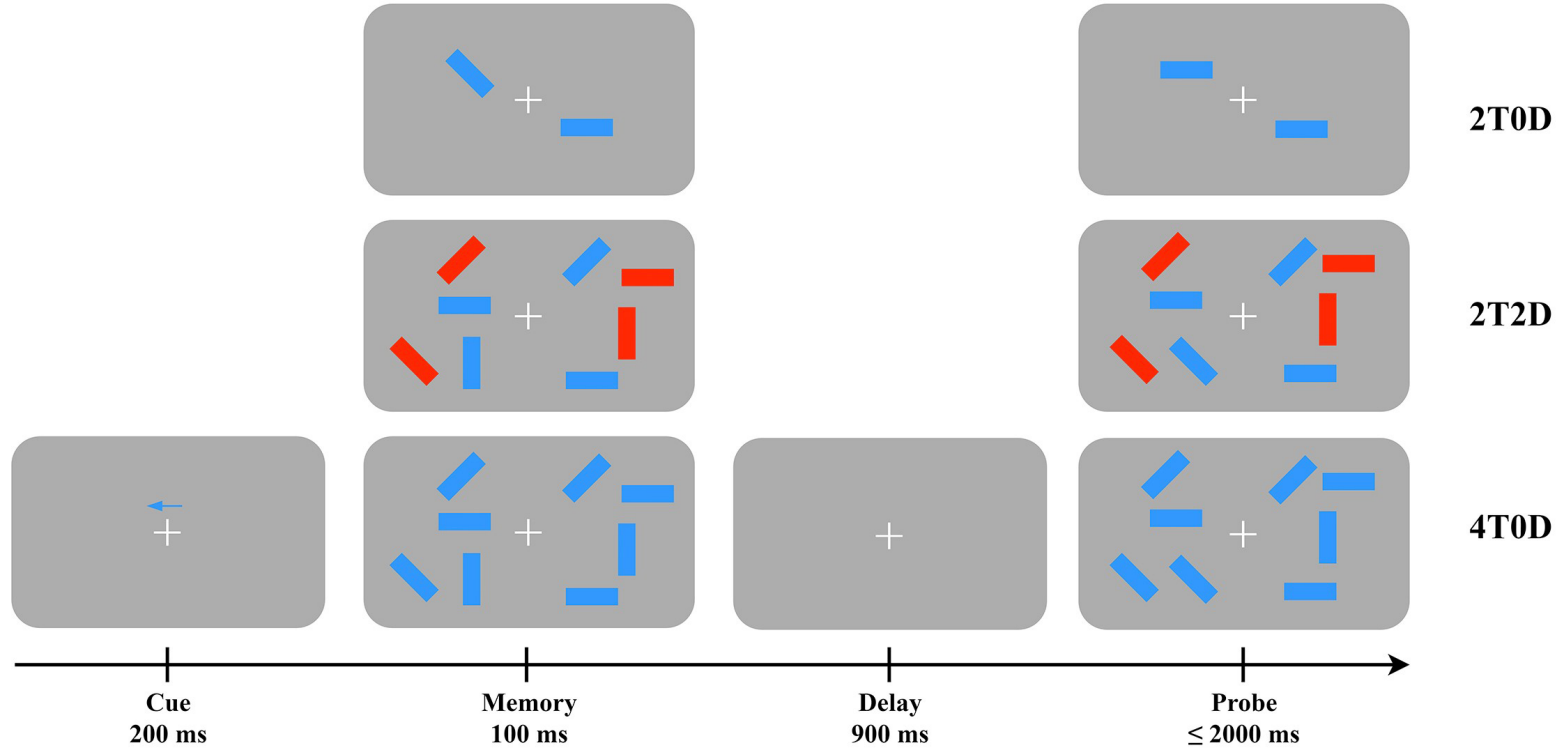
**Flowchart of the change detection task**. Blue Rectangles: the core target stimuli to be remembered. Their orientation (arrow-like direction) is the primary feature participants monitor for changes. Red Rectangles: distractor stimuli (only present in the 2T2D condition). They introduce a secondary feature dimension (color + orientation) to increase task complexity and test memory for feature conjunctions. 2T0D: two targets, zero distractors. This is a low-load, single-feature (orientation only) condition. 2T2D: two targets, two distractors (red rectangles). This is a dual-feature (color + orientation) condition that tests binding of visual features in memory. 4T0D: four targets, zero distractors. This is a high-load, single-feature condition used to assess visual working memory capacity limits. Fixation point (+): a central fixation cross presented at the center of the screen. Participants were required to keep their eyes fixated on this symbol and avoid gaze drift during stimulus presentation.

The experimental paradigm utilized the change detection task. The stimulus material presented to participants consisted of red or blue color targets appearing on both sides of fixation in various orientations (0°, 45°, 90°, 135°), with the color targets displayed in random orientations. The experimental program was developed and executed using E-Prime 3.0 (Psychology Software Tools (PST), Inc., Pittsburgh, PA, USA). At the beginning of each trial, a spatial arrow cue was presented for 200 ms, prompting participants to remember the target stimulus associated with the specified visual field side (left, right) and color (red, blue). This was followed by a 100 ms memorization page, immediately succeeded by a 900 ms delay page. Finally, a probe page was displayed, where participants were required to quickly and accurately determine whether the direction of the target stimulus had changed for the specified visual field side and color (with a maximum response time of 2000 ms; see Fig. 1). The task comprised three conditions: two targets, zero distractors (2T0D), two target stimuli presented simultaneously with two distractor stimuli (2T2D), and four targets, zero distractors (4T0D). In the formal experiment, there were 80 trials for each condition, resulting in a total of 240 trials.

### 2.4 EEG Data Acquisition and Data Preprocessing

The experimental apparatus consisted of a 64-channel EEG/ERP system from NeuroScan (Neuroscan Compumedics Limited, Abbotsford, Victoria, Australia). The right mastoid (M2) served as the reference electrode during data acquisition, while the grounding electrode (FPZ) was positioned at the center of the forehead. Vertical eye movement electrodes (VEOG) were placed 2 cm above and below the left orbit, and horizontal eye movement electrodes (HEOG) were positioned approximately 1 cm lateral to the outer canthus of both eyes. A sampling frequency of 1000 Hz was selected, and a bandpass filter ranging from 0.01 to 100 Hz was applied to ensure that the impedance between the scalp and the electrodes remained below 10 kΩ.

EEG data analysis was performed offline using the EEGLAB2024 (https://sccn.ucsd.edu/eeglab/) [38]. The data were re-referenced to the average of all electrodes and downsampled to 256 Hz. EEG data were band-pass filtered between 0.1 and 20 Hz using an infinite impulse response (IIR) filter with a roll-off rate of 12 dB/octave [39]. EEGLAB includes an independent component analysis (ICA) method to correct eye movement artifacts and remove ICA components based on automated criteria. In the PSZ group, the mean number of removed ICA artifact components was 5.64 ± 1.73. In the HCS group, the mean number was 6.33 ± 2.43. The data were then segmented to mark the onset of the memory stimulus, with a time window extending from 200 ms before to 1000 ms after the stimulus presentation. The interval from –200 to 0 ms relative to the stimulus was selected as the baseline. Rejects other noise artifacts with amplitudes exceeding ±100 µV. Ultimately, the mean number of trials was 72.95 ± 5.91 in the PSZ group and 74.17 ± 5.25 in the HCS group.

Possible sources of the CDA have been identified in the posterior parietal cortex [20, 21, 22]. For this study, three representative pairs of parieto-occipital electrodes (PO3, PO4, PO5, PO6, PO7, PO8) were selected. The choice of electrode sites was informed by prior research that utilized CDA as an indicator of vWM capacity [40, 41]. The CDA wave amplitude was calculated by subtracting the average voltage of the electrodes in the contralateral hemisphere from the average voltage of the electrodes in the ipsilateral hemisphere corresponding to the memory side [25]. In this study, the CDA was computed by averaging the wave amplitude from 300 to 800 ms following stimulus presentation, with the interval selection based on previous findings [42, 43].

### 2.5 Decoding Analyses

The preprocessed ERP data were decoded and analyzed using ERPLAB 10.0 (https://erpinfo.org/erplab) with an SVM classifier to perform MVPC [44]. Decoding accuracy for individual participants was calculated using 5-fold cross-validation with 100 iterations. Finally, the average decoding accuracy for each group of participants was computed. We utilized 60 active electrodes as features for decoding. Since we decoded both memory load (2T0D/2T2D/4T0D) and memory side (left/right), the chance level was 1/6. Only accuracy could be reported, and no additional metrics could be provided.

In addition, to further investigate the contribution of individual electrode data to inter-conditional decoding accuracy, we selected data from the CDA time window (300–800 ms) for searchlight decoding analysis, utilizing the MVPA-Light package in MATLAB R2021b (https://www.mathworks.com/) [45], with LIBSVM as the classifier. For each EEG channel, we identified adjacent channels with spatial distances of less than 60 mm to form local clusters [46], and then conducted decoding analysis on these channels (median = 10 electrodes per cluster; minimum = 5, maximum = 13).

### 2.6 Data Analyses

vWM capacity is calculated using Cowan’s K [47], where K=(H-F⁢A)×N. In this formula, H (Hit rate) represents the probability that a participant responds when a change occurs, FA (False Alarm rate) indicates the probability of responding when no change occurs, and N (Set size) denotes the memory load.

Statistical analysis was primarily conducted using SPSS (v26.0, IBM Corp., Armonk, NY, USA). Demographic data and scale scores for the PSZ and HCS groups were compared using independent samples *t*-tests or chi-square tests. Behavioral indicators and CDA amplitudes were analyzed between the two groups using repeated-measures analysis of variance (ANOVA). Differences in decoding accuracy and chance probability were compared using one-sample *t*-tests. Decoding accuracy between the two groups of subjects was compared using independent samples *t*-tests. A false discovery rate (FDR) correction was applied to control for multiple comparison bias. A two-sided test was conducted with a significance level of α = 0.05.

To assess the relationship between working memory capacity, CDA amplitude, and decoding accuracy, correlation analyses were conducted on the indices for attention scope, indices for attention control, ΔCDA for scope, ΔCDA for control, and mean MVPC accuracy (300–800 ms). The indices for attention scope are defined as $\text{max}\,\left(K_{\text{2T0D}},K_{\text{4T0D}}\right)$, while the indices for attention control are calculated as $2+\left(K_{\text{2T2D}}-K_{\text{2T0D}}\right)$ [48]. The ΔCDA for scope is determined by $C\,D\,A_{\text{4T0D}}-C\,D\,A_{\text{2T0D}}$, and the ΔCDA for control is calculated as $C\,D\,A_{\text{2T2D}}-C\,D\,A_{\text{2T0D}}$ [49]. Spearman’s correlation analysis was employed due to the non-normal distribution of the data, with FDR correction applied to control for multiple comparison bias. A two-sided test was conducted with a significance level of α = 0.05.

## 3. Results

### 3.1 Demographics and Scale Results

Between-group comparisons of the PSZ and HCS revealed no significant differences in gender, age, or education levels. Independent samples *t*-tests conducted on the participants’ scores for the spatial breadth test and the number sequence test indicated a significant group main effect for the spatial breadth test, with the PSZ group scoring lower than the HCS group (t(44) = 4.91, *p* < 0.001). Conversely, for the number sequence test, there was also a significant group main effect, with the PSZ group scoring lower than the HCS group (t(44) = 4.44, *p* < 0.001) (Table 1). Thus, the PSZ group demonstrated a lower working memory capacity compared to the HCS group.

**Table 1.** **Demographic information and scale results for the PSZ and HCS groups**.

	PSZ (Mean ± SD)	HCS (Mean ± SD)	t/χ^2^	*p*
Age (years)	29.23 ± 7.04	31.60 ± 5.25	1.32	0.20
Sex (M/F)	13/9	13/11	0.11	0.74
Education (years)	12.45 ± 3.20	12.10 ± 3.08	–0.36	0.72
MCCB - Spatial Span	11.91 ± 3.34	16.88 ± 3.51	4.91	<0.001
MCCB - Number Span	11.43 ± 2.70	15.42 ± 3.33	4.44	<0.001
Course (years)	6.02 ± 4.45	-	-	-
PANSS – General	25.23 ± 6.38	-	-	-
PANSS – Positive	12.50 ± 5.54	-	-	-
PANSS – Negative	17.05 ± 9.62	-	-	-
PANSS – Total	54.77 ± 11.93	-	-	-

PSZ, patients with schizophrenia; HCS, healthy controls; MCCB, MATRICS Consensus Cognitive Battery; PANSS, Positive and Negative Syndrome Scale; M, male; F, female.

### 3.2 Behavioural Results

**Fig. 2.**
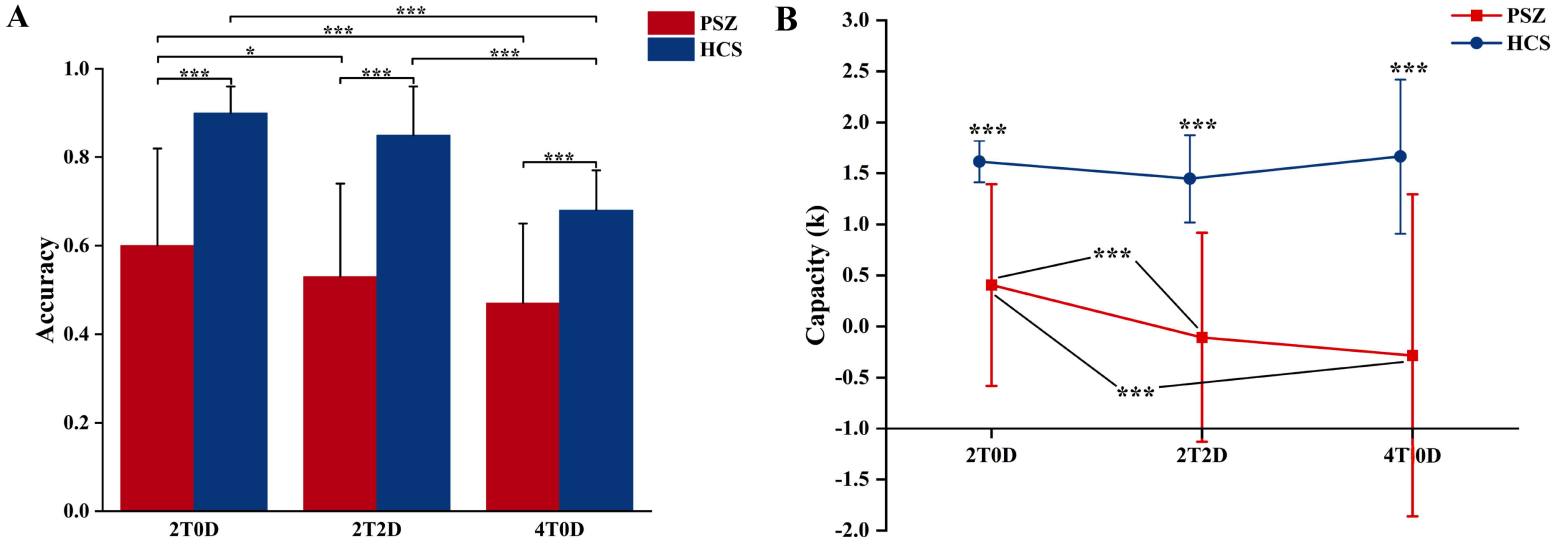
**Behavioural results**. (A) Accuracy of the PSZ and HCS groups under different memory loads. The error bars represent the mean ± standard deviation. (B) K values of the PSZ and HCS groups under different memory loads. The error bars represent the mean ± standard deviation. *** indicates *p* ≤ 0.001, and * indicates *p* ≤ 0.05.

A two-factor repeated-measures ANOVA was conducted to assess accuracy. The main effect of group was significant, with the PSZ group demonstrating significantly lower accuracy compared to the HCS group (F(1, 44) = 44.57, $\eta_{p}^{2}$ = 0.50, *p* < 0.001). The main effect of memory load was also significant (F(2, 88) = 48.50, $\eta_{p}^{2}$ = 0.52, *p* < 0.001). Post hoc multiple comparisons revealed that the 2T0D condition was more accurate than the 2T2D condition (*p* = 0.006), and the 2T2D condition was more accurate than the 4T0D condition (*p* < 0.001). The interaction between group and memory load was significant (F(2, 88) = 5.34, $\eta_{p}^{2}$ = 0.11, *p* = 0.01). Further simple effects analyses indicated that the PSZ group was significantly less accurate than the HCS group across all three memory loads (*p* < 0.001). The PSZ group demonstrated significantly higher accuracy in 2T0D compared to 2T2D (*p* = 0.02) and in 2T2D compared to 4T0D (*p* < 0.001). Similarly, the HCS group exhibited significantly greater accuracy in 2T0D than in 4T0D (*p* < 0.001) and in 2T2D compared to 4T0D (*p* < 0.001). The other conditions did not show significant differences (Fig. 2A).

A two-way repeated-measures ANOVA was conducted on K. The main effect of group was significant, with the PSZ group exhibiting significantly lower K values than the HCS group (F(1, 44) = 43.23, $\eta_{p}^{2}$ = 0.50, *p* < 0.001). The main effect of memory load was also significant (F(2, 88) = 5.51, $\eta_{p}^{2}$ = 0.11, *p* = 0.01). Post hoc multiple comparisons indicated that the K values for the 2T0D condition were significantly higher than those for the 2T2D condition (*p* < 0.001) and also higher than those for the 4T0D condition (*p* = 0.04). However, the K values for the 2T2D condition did not differ significantly from those for the 4T0D condition (*p* = 1.000). The interaction between group and memory load was also significant (F(2, 88) = 5.17, $\eta_{p}^{2}$ = 0.11, *p* = 0.01). Further simple effects analyses revealed that the patient group exhibited significantly lower K values than the healthy group across all three memory loads (*p* < 0.001). Additionally, the patient group demonstrated significantly higher K values for the 2T0D condition compared to the 2T2D condition (*p* < 0.001) and higher K values for the 2T0D condition compared to the 4T0D condition (*p* = 0.001). There was no significant difference between the 2T2D and 4T0D conditions (*p* = 1.00), and none of the remaining conditions showed significant results (Fig. 2B).

### 3.3 CDA Results

**Fig. 3.**
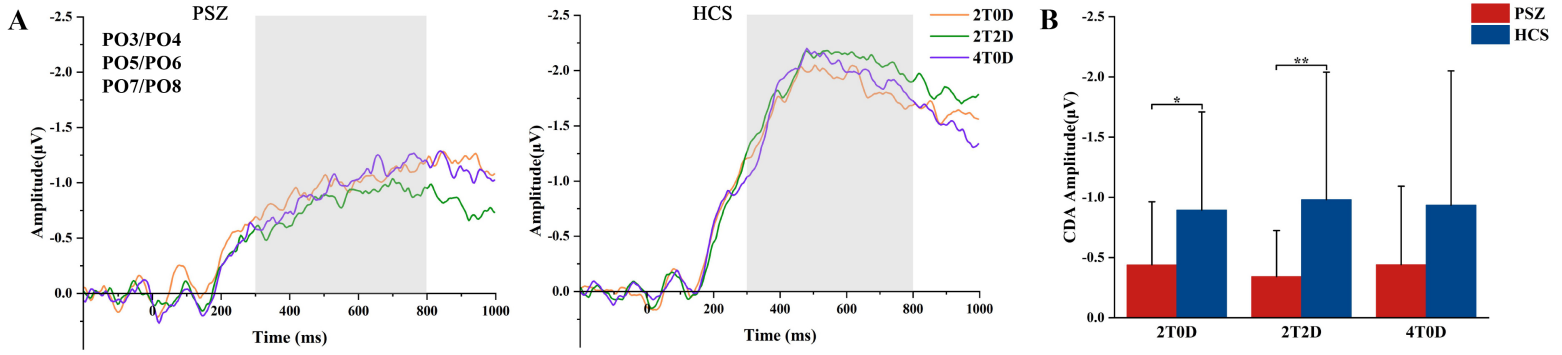
**CDA results**. (A) CDA wave amplitudes (PO3/PO4, PO5/PO6, PO7/PO8) in the PSZ and HCS groups under different memory loads, with gray rectangles indicating the CDA time windows. (B) Mean CDA wave amplitudes (300–800 ms) in the patient and healthy groups across different memory loads; * indicates *p* ≤ 0.05, ** indicates *p* ≤ 0.01. CDA, contralateral delay activity; PO, parieto-occipital electrodes.

A two-factor repeated measures ANOVA was conducted on CDA amplitude (Fig. 3). The results indicated a significant main effect of group, with the PSZ group exhibiting significantly lower CDA amplitude than the HCS group (F(1, 44) = 5.63, $\eta_{p}^{2}$ = 0.11, *p* = 0.02). The main effect of memory load was not significant (F(2, 88) = 0.07, $\eta_{p}^{2}$ = 0.002, *p* = 0.94). The interaction between group and memory load was not significant (F(2, 88) = 0.81, $\eta_{p}^{2}$ = 0.02, *p* = 0.45). Further analyses demonstrated that the CDA amplitude in the PSZ group was significantly smaller than that in the HCS group at a memory load of 2T0D (*p* = 0.03) and 2T2D (*p* = 0.01).

### 3.4 Decoding Results

**Fig. 4.**
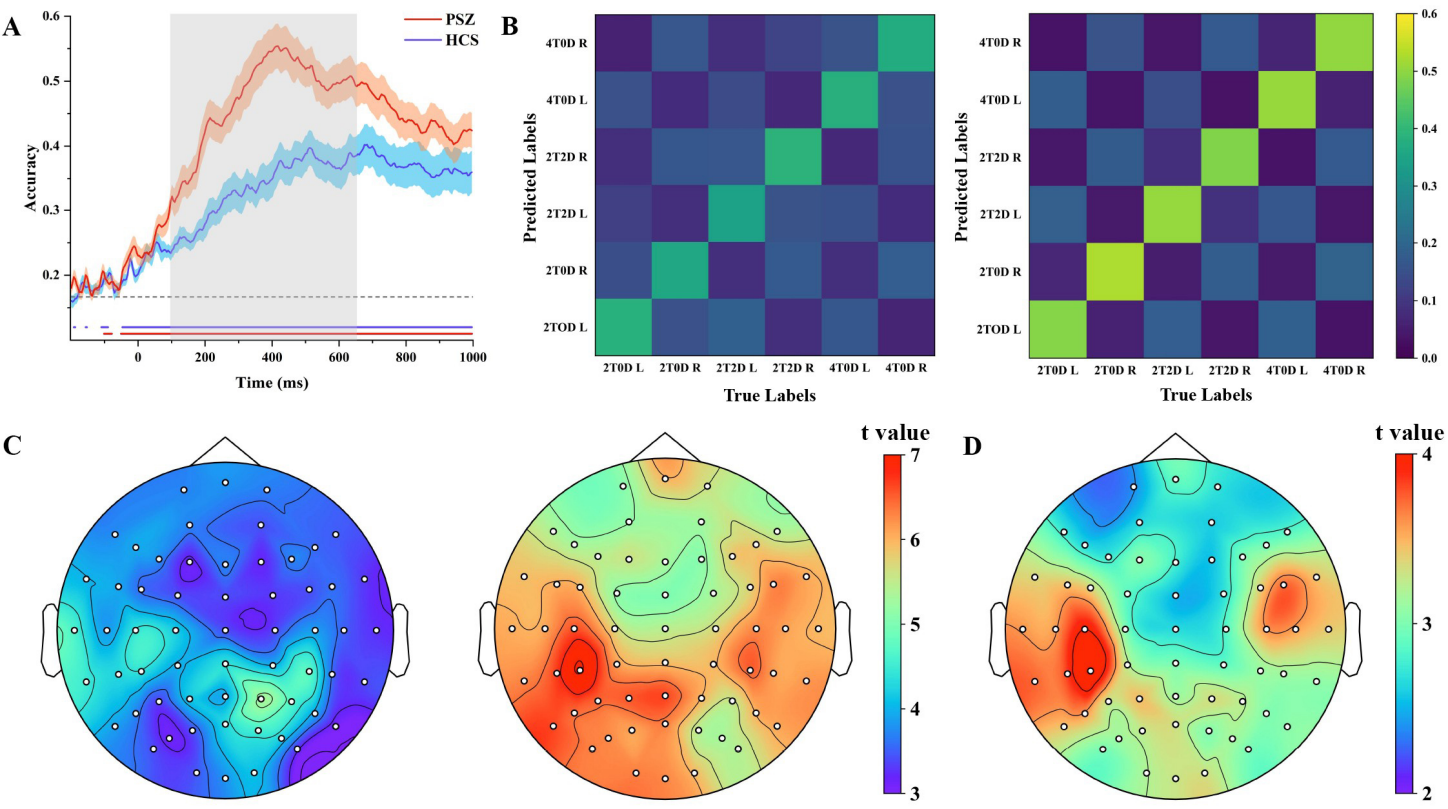
**Decoding accuracy results**. (A) Trends in decoding accuracy over time in the PSZ and HCS groups. The gray dashed lines indicate the chance level, while the red and blue solid lines mark the time points at which the PSZ and HCS groups significantly exceeded this chance level. The gray matrices highlight the time points where the decoding accuracy for the PSZ group was significantly lower than that of the HCS group. (B) MVPC accuracy confusion matrices (300–800 ms) for both groups, with L denoting the left memory side and R denoting the right memory side. (C) Electrode channels influenced by the PSZ and HCS groups during the task, with all electrode sites showing significant activation. (D) Results of the significance test for the searchlight accuracy of each electrode site in both the PSZ and HCS groups, revealing that all electrode sites exhibited significant differences. MVPC, multivariate pattern classification.

MVPC accuracy and chance level were compared using one-sample *t*-tests. As shown in Fig. 4A, the results indicated that in the PSZ group, the whole-brain ERP spatial distribution effectively distinguished among stimulus categories within a time window of –50 to 1000 ms (*p*_corrected_ < 0.05). In the HCS group, the whole-brain ERP spatial distribution effectively distinguished among stimulus categories within a time window of –46 to 1000 ms (*p*_corrected_ < 0.05). Independent samples *t*-tests were conducted to assess MVPC accuracy. The results indicated that the decoding accuracy in the PSZ group was significantly lower than that in the HCS group from 93 to 652 ms (*p*_corrected_ < 0.05). The confusion matrix of MVPC accuracy within the CDA time window revealed that, in the HCS group, MVPC accuracy tended to increase as memory load increased (Fig. 4B).

A one-sample *t*-test was conducted to compare searchlight accuracy with the chance level. The results indicated that the whole-brain electrodes successfully discriminated among stimulus categories in both the PSZ and HCS groups (*p*_corrected_ < 0.05, Fig. 4C). An independent samples *t*-test for searchlight accuracy revealed that decoding accuracy was significantly lower in the PSZ group compared to the HCS group across all electrode sites (*p*_corrected_ < 0.05), with the largest difference observed in the left parietal electrodes (Fig. 4D).

### 3.5 Correlation Results

**Fig. 5.**
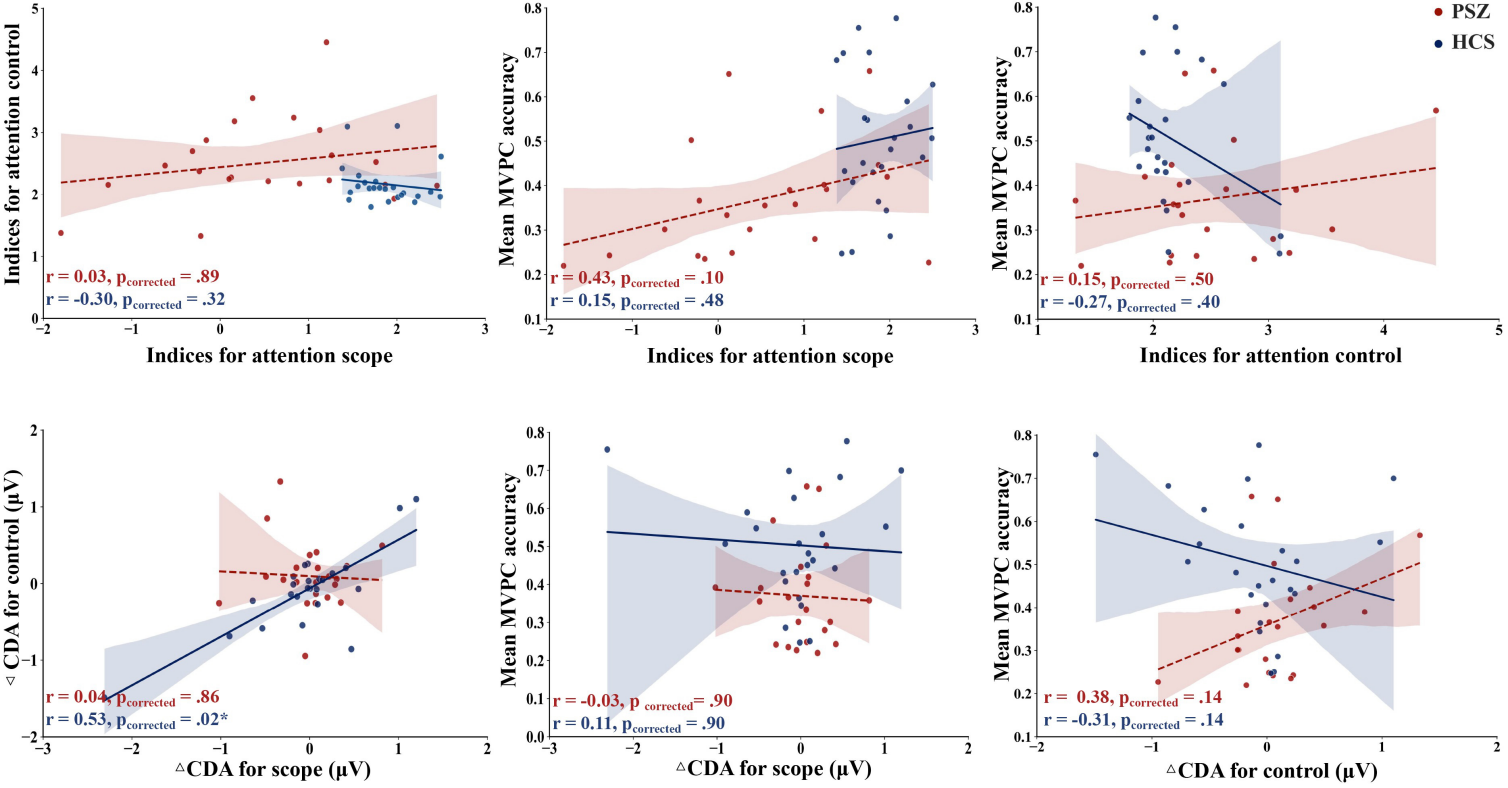
**Correlation results**. The red dashed line represents the linear regression fit for the PSZ group, while the blue solid line represents the linear regression fit for the HCS group. The shaded areas indicate the 95% confidence interval of the fit. In the HCS group, ΔCDA for scope demonstrated a significant positive correlation with ΔCDA for control. * indicates *p* ≤ 0.05.

As illustrated in Fig. 5, correlation analyses indicated that in the HCS group, ΔCDA for scope demonstrated a significant positive correlation with ΔCDA for control (r = 0.53, *p* = 0.02). None of the other correlations reached statistical significance.

## 4. Discussion

In this study, we employed decoding methods using ERPs to investigate the differences in neural representations between PSZ and HCS during a change detection task. (1) The MVPC results indicated that ERPs from both the PSZ and HCS groups successfully decoded memory load and memory side information. However, compared to HCS, the PSZ group exhibited lower decoding accuracy during the delay period (93–652 ms), suggesting that the ERPs of the PSZ group contained less stimulus-related information. (2) The searchlight analysis revealed that brain regions of both PSZ and HCS groups were fully activated during the change detection task, with the parieto-occipital region being particularly sensitive to the stimulus category. In terms of whole-brain electrode data, the decoding accuracy for the PSZ group was lower than that of the HCS group, especially in the left parietal electrodes. In conclusion, decoding methods can be effectively applied at the individual subject level to enhance our understanding of the nature of impaired cognitive functioning in individuals with PSZ.

The MVPC results indicated that the decoding accuracy of both PSZ and HCS was significantly higher than chance level, consistent with findings from previous studies. Bae *et al*. [29] employed the decoding method to evaluate the information content of PSZ ERPs and discovered that the decoding accuracy of PSZ regarding memory side (left/right) exceeded chance level across all three types of memory load (1/3/5T). In the present study, both memory load and memory side were decoded simultaneously, and the decoding accuracy of PSZ and HCS was substantially above chance level. Decoding analyses were conducted for each participant individually, with training and testing based on the means of different trial subsets. The decoding accuracy reflects the ability of each participant’s neural signals to reliably predict the stimulus information to be remembered. Therefore, during the change detection task, the ERPs of both PSZ and HCS contained a sufficient amount of information regarding stimulus categories. It is noteworthy that the decoding accuracy was higher than chance even before the stimulus appeared. This may be because, prior to presenting the stimuli to be memorized, subjects were shown 100 ms of cue information indicating the designated side of the visual field to be memorized. Future studies could consider performing decoding analysis under conditions where cue and stimulus information are presented simultaneously to prevent information leakage.

Compared to HCS, PSZ exhibited lower MVPC accuracy during the delay period, indicating that the ERPs of PSZ contained less information regarding stimulus categories. EEG studies that elicited ERPs during a WM task revealed abnormal electrical activity during both early evoked responses and late cognitively relevant components in the PSZ [50]. Numerous investigations into the neural mechanisms underlying WM deficits in the PSZ have demonstrated significant neural inefficiency, characterized by functional hypoconnectivity within frontoparietal networks [51, 52] and diminished functional interactions between large-scale networks [53, 54]. This may result in decreased efficiency of PSZ in encoding neural information during the delay period, leading to a reduction in the information content of ERPs.

Searchlight analysis revealed that whole-brain electrodes from both PSZ and HCS could significantly discriminate between stimulus categories, with the parieto-occipital region demonstrating the highest sensitivity to these categories. Across the electrode sites, the decoding accuracy for PSZ was notably lower than that for HCS, particularly in the left parietal region, which was especially significant. A recent study indicated that reduced functional connectivity capacity (rFCS) in the left subparietal region is a neurophysiological characteristic that differentiates PSZ from HCS [55]. Previous research has also identified abnormalities in the left subparietal cortex of PSZ, where atypical cortical modulation has been linked to deficits in memory, audiovisual integration, and emotional processing [56]. Furthermore, resting-state EEG micromorphological changes in PSZ exhibited hyperactivation of the left subparietal lobule and a reduction in resting-state micromap duration [57]. Consequently, we hypothesized that during the vWM task, PSZ would exhibit less stimulus information in the ERP signal due to the abnormalities in the left parietal region, resulting in particularly lower decoding accuracy for the left parietal electrode compared to HCS.

Numerous studies have demonstrated an increase in posterior parietal CDA amplitude with an expanding attention scope in vWM [58, 59, 60, 61, 62, 63, 64]. A recent investigation revealed that the processes of attention control and attention scope share similar posterior parietal CDA characteristics [48]. The present study replicated this finding, showing that in the HCS, the 2T2D condition induced a larger CDA amplitude compared to the 2T0D condition. Furthermore, individuals in the HCS group who exhibited greater wave amplitudes in the 4T0D condition also demonstrated higher wave amplitudes in the 2T2D condition. These results support the notion that attention control elicits CDA wave amplitudes comparable to those associated with attention scope. In PSZ, the 2T2D condition necessitates the combined engagement of attention scope and attention control. However, due to significant attention deficits, the CDA wave amplitude in the 2T2D condition is notably smaller than that observed in the healthy group.

Although the present study successfully revealed the spatiotemporal dynamics underlying vWM deficits in PSZ, several limitations remain. The main effect of memory load on the CDA index was not significant. This may be attributed to the introduction of interference stimuli in this study, which aimed to investigate the differences between scope and control processes in working memory. In HCS, the CDA amplitude was slightly higher in the 4T0D condition compared to the 2T0D condition, but this difference did not reach statistical significance. Additionally, Zhang *et al*. [48] identified a significant positive correlation between the indices of attention scope and attention control. However, in the present study, no significant correlation was found. This lack of correlation may be attributed to the small sample size and the heterogeneity of participants. Finally, since we used ERPLAB for MVPC decoding analysis, the method provides only accuracy as an outcome metric and does not include other metrics, such as the F1 score or balanced accuracy. While accuracy provides a quick overview of overall correctness, it does not fully capture differences in model performance across categories. Especially in unbalanced datasets, where accuracy often provides overly optimistic estimates of model performance [65]. Future studies should aim to increase sample sizes and incorporate more comprehensive outcome measures to achieve more robust results.

## 5. Conclusions

In conclusion, this study investigates the differences in ERP neural representations between PSZ and HCS in vWM. We employed decoding methods to reveal the spatiotemporal dynamics of vWM deficits in PSZ. The findings indicate that information-based decoding methods can offer valuable insights into the neural representation of information in specific populations while they perform a task.

## Availability of Data and Materials

The data that support the findings of this study are available on request from the corresponding author.
